# Non-destructive Measurements of *Toona sinensis* Chlorophyll and Nitrogen Content Under Drought Stress Using Near Infrared Spectroscopy

**DOI:** 10.3389/fpls.2021.809828

**Published:** 2022-01-21

**Authors:** Wenjian Liu, Yanjie Li, Federico Tomasetto, Weiqi Yan, Zifeng Tan, Jun Liu, Jingmin Jiang

**Affiliations:** ^1^Research Institute of Subtropical Forestry, Chinese Academy of Forestry, Hangzhou, China; ^2^AgResearch Ltd., Christchurch, New Zealand; ^3^Department of Computer Science, Auckland University of Technology, Auckland, New Zealand

**Keywords:** NIR spectroscopy, drought stress, chlorophyll and nitrogen contents, variable selection, dynamic monitoring, partial least square regression (PLSR)

## Abstract

Drought is a climatic event that considerably impacts plant growth, reproduction and productivity. *Toona sinensis* is a tree species with high economic, edible and medicinal value, and has drought resistance. Thus, the objective of this study was to dynamically monitor the physiological indicators of *T. sinensis* in real time to ensure the selection of drought-resistant varieties of *T. sinensis*. In this study, we used near-infrared spectroscopy as a high-throughput method along with five preprocessing methods combined with four variable selection approaches to establish a cross-validated partial least squares regression model to establish the relationship between the near infrared reflectance spectroscopy (NIRS) spectrum and physiological characteristics (i.e., chlorophyll content and nitrogen content) of *T. sinensis* leaves. We also tested optimal model prediction for the dynamic changes in *T. sinensis* chlorophyll and nitrogen content under five separate watering regimes to mimic non-destructive and dynamic detection of plant leaf physiological changes. Among them, the accuracy of the chlorophyll content prediction model was as high as 72%, with root mean square error (RMSE) of 0.25, and the RPD index above 2.26. Ideal nitrogen content prediction model should have *R*^2^ of 0.63, with RMSE of 0.87, and the RPD index of 1.12. The results showed that the PLSR model has a good prediction effect. Overall, under diverse drought stress treatments, the chlorophyll content of *T. sinensis* leaves showed a decreasing trend over time. Furthermore, the chlorophyll content was the most stable under the 75% field capacity treatment. However, the nitrogen content of the plant leaves was found to have a different and variable trend, with the greatest drop in content under the 10% field capacity treatment. This study showed that NIRS has great potential for analyzing chlorophyll nitrogen and other elements in plant leaf tissues in non-destructive dynamic monitoring.

## Introduction

Due to global climate change, droughts around the world have become more frequent and have increased in severity, which will have a serious impact on the growth of plants and crops (Stocker, 2014; Mazis et al., 2020). In addition, extreme drought and a lack of precipitation are thought to exacerbate climate change (Jia et al., 2020). Drought impacts vegetation differentially across fields, seasons and species (Douma et al., 2012), and available water ground and rain are the most important factors that significantly influence plant growth and productivity (Hoover et al., 2014; Gao et al., 2019; Khaleghi et al., 2019). The reduction in groundwater leads to potential plant mortality (Estiarte et al., 2016). Recently, more attention has been given to how plants respond to water availability (Khaleghi et al., 2019). Drought resistance (DR) is defined as the mechanism causing minimum water loss in a water deficit environment while maintaining its production. DR is determined by how quickly and efficiently a plant senses changing environmental conditions, and how the plant adopts and combines the aforementioned strategies in response to diminished water availability (Baresel et al., 2017). DR is linked to a combination of morphological, anatomical and physiological traits (Lozano et al., 2020). In fact, plant species in dry environments have deeper roots, slightly denser stems, thicker and denser leaves and relatively high N content per leaf space to optimize water usage (Markesteijn et al., 2011). Conversely, drought stress typically reduces photosynthetic capacity and carbon storage in the form of non-structural carbohydrate (NSC) concentrations and plant respiration rates (Centritto et al., 2009; Bongers et al., 2017).

*Toona sinensis*, also called Chinese toon or Chinese mahogany, is a deciduous woody plant, with straight trunk, hard wood and beautiful texture, has high economic value in the wood industry (Peng et al., 2019). In addition, *T. sinensis* is also a precious medicinal plant as the leaves rich in protein, fat, minerals, flavonoids, terpenoids, and vitamins (Chen et al., 2009; Shi et al., 2021). However, the nutrient element in the leaves varies rapidly under the influence of water, with DR (Peng et al., 2019).

The leaf is a vital organ for measuring plant ecological traits (Petit Bon et al., 2020). Plants under drought conditions will reduce leaf area and increase leaf thickness (Rowland et al., 2020). Drought stress also alters plant physiological processes, among which amendments to pigment composition and subsequent photosynthesis are the most critical (Males and Griffiths, 2017). A reduction in chlorophyll affected by moisture content has been reported (He and Dijkstra, 2014). The chlorophyll content incorporates a sensible correlation with the photosynthetic capacity and the development stage of vegetation, which are indicators of photosynthetic capacity (Zhang et al., 2014). Nitrogen (N) is one of the main macroelements needed for plant growth. Nitrogen in plants constitutes amino acids and proteins, but it is also an elementary component of chlorophyll nucleic acids, multiple coenzymes, vitamins, and plant hormones (Hammad and Ali, 2014). N plays a crucial role in evaluating the intensity of vegetation photosynthesis and vegetation nutritional status. Therefore, the chlorophyll and nitrogen content of plant leaves can be used to analyze DR.

Traditionally, chemical methods in the laboratory are used to detect physiological signals. Although highly accurate, these methods are destructive, time-consuming, expensive, and use highly contaminating reagents. Near infrared reflectance spectroscopy (NIRS) has recently been found to provide cost-effective and accurate measures of chemical traits in plant leaves regardless of species, ecological environment or region. NIRS is rapid, chemical-free, simple to use and non-destructive (Manley, 2014). Previous work with *Medicago sativa* (Naya et al., 2007) demonstrated that NIRS were able to measure macronutrients and micronutrients. Relative reflectance is the near infrared range (between 800 and 1,200 nm) and was also used to show drought-related stress impacts (Mazis et al., 2020). Numerous research investigations have used NIRS to predict the ecophysiological variables related to plant drought stress, which constrains relative water and leaf water in disease-resistant trees (Warburton, 2014; Conrad and Bonello, 2016). Partial least squares regression (PLSR) is one of the most frequently used chemometric methods in spectral calibration analysis (Li et al., 2021). It has advantages when large amounts of data with redundancy and high collinearity exist and when the number of variables is greater than the number of samples (Liang et al., 2020). Pre-processing approaches like standard normal variate (SNV), smoothing and derivatives are often required in the process of spectral analysis (Li et al., 2021). In addition, we can also choose a variety of variable selection methods to reduce the impact of irrelevant variables on the accuracy of the model (Shao et al., 2017). However, due to strict spectral models, plant species and their spectral measurement required various assumptions for the proposed model.

In our study, three varieties of *T. sinensis* seeds with large differences in DR were selected as experimental materials. For the first time, we used NIRS to predict nutrient content in *T. sinensis* leaves in a drought stress environment and dynamically monitor the drought response of *T. sinensis* seedlings in real time to ensure the selection of high-quality drought-resistant varieties of *T. sinensis*. Here, three hypotheses were proposed: (1) the PLSR model combined with preprocessing methods and four variable selection methods could predict the chlorophyll and nitrogen content of *T. sinensis* leaves; (2) NIRS bands could characterize wavelengths related to chlorophyll and nitrogen in *T. sinensis* leaves; and (3) NIRS models could detect chlorophyll and nitrogen content in *T. sinensis* under various drought stresses.

## Materials and Methods

### Plant Material and Treatments

The experiment was conducted in a greenhouse on a mountain in Fuyang, Zhejiang, China (E 119.57′, N 30.03′). The annual average temperature in the greenhouse is 28°C, with relative humidity > 75% and daily sunshine up to 13 h, making growth conditions very suitable for *T. sinensis* seedlings. To study the differences in DR between different *T. sinensis* varieties, three varieties of *T. sinensis* seeds with large variations in DR from northern (N), central (C) and southern (S) China were selected as experimental materials.

In the initial stage, seedlings of relatively consistent size were selected from the nutrient cup [16 cm (d) × 14 cm (h)] and transplanted into the experimental pot [30 cm (h) × base 27 cm (d)]. An appropriate amount of compound fertilizer was applied uniformly, leaving the seedlings to grow for approximately 14 days. When the seedlings grew five to seven functional leaves, the water control treatment started (August 10, 2020).

### Experimental Design

A completely randomized experimental design was conducted in a greenhouse to model various physiological traits of *T. sinensis* leaves under various water stresses. Five water gradients were created: (I) in the control treatment, where the pots were watered to 100% field capacity (FC) replacing the amount of water transpired daily (100% FC); (II) light stress (75% FC) with relative soil water content (RWC) accounting for field holding capacity with 75% of water content; (III) moderate severe stress (50% FC) with RWC accounting for 50% of field water holding capacity; (IV) severe stress (30% FC) with RWC accounting for field holding capacity of 30% of water content; (V) extreme stress (10% FC) with RWC accounting for 10% of field water holding capacity (Khaleghi et al., 2019).

As shown in Table 1, there were two blocks in this experiment. Block 1 was used for model construction. There were 20 *T. sinensis* seedlings of three varieties in each treatment, for a total of 300 seedlings. The basin weighing method for soil was adopted for moisture control of the soil within the set range, weighing it once every 3 days, and replenishing water occasionally. To ensure the accuracy and stability of the model, the spectrum was collected at 3 pm every Saturday, followed by destructive sampling to measure chlorophyll and nitrogen indicators. For each treatment, up to three *T. sinensis* seedling varieties were selected, and then six leaves were selected from the upper, middle and lower parts of each plant for spectral measurements. After collecting the spectrum, the corresponding *T. sinensis* leaves were picked, numbered and placed in a paper bag and then sent back to the laboratory in a 4°C freezer for refrigeration to measure the chlorophyll and nitrogen content (Li et al., 2019). The first data collection and trait measurement began after the first week of water control. Repeat the above operation 6 times. Block 2 was used for model verification, dynamically monitoring the changes in chlorophyll and nitrogen content of *T. sinensis* seedlings under different periods and various drought treatments. In this experiment, *T. sinensis* seedlings of each variety and in each treatment (*n* samples = 10) were selected (total of 150 samples). Every Saturday at 5 pm, the upper, middle, and lower parts of the plant were selected to collect corresponding near-infrared spectroscopy information using the same method. The experiment lasted for 2 months.

**TABLE 1:** The number and layout of *T. sinensis* seedlings from northern (N), central (C), and southern (S) China under different treatment conditions.

	100%FC			75%FC			50%FC			30%FC			10%FC		
	N	C	S	N	C	S	N	C	S	N	C	S	N	C	S
Block 1	20	20	20	20	20	20	20	20	20	20	20	20	20	20	20
Block 2	10	10	10	10	10	10	10	10	10	10	10	10	10	10	10

### Near Infrared Reflectance Spectroscopy Collection

The NIRS data were taken from the upside surface of the leaves three times with a handheld fiber optic contact probe from a field-based spectrometer (LF-2500, Spectral Evolution, United States) (Li et al., 2021). Each spectrum took on average 20 scans with 8 m/s integration time and a range between 1,100 and 2,500 nm with a 6 nm spectral resolution. In total, 760 samples were collected for the construction of chlorophyll (*n* = 360) and nitrogen (*n* = 400) content prediction models.

### Leaf Chlorophyll Content Measurement

A mixed solution of 5 ml 1:1 (5 ml acetone: 5 ml absolute ethanol) was added to a test tube. We took 0.5 g of *T. sinensis* leaves, cut them into one mm wide filaments and put the sample into a test tube. We then sealed the test tube and placed it in the dark to soak for 24 h. For the chlorophyll measurement, we took one ml of extract sample and two ml of a mixture of acetone and pure ethanol. We then used a UV–visible spectrophotometer (UV-1280, Shimadzu, Japan) to measure chlorophyll absorbance at 645 and 663 nm (Gu et al., 2016).

The following formula were applied:


$$\mathrm{Chlorophyll}\,a=\,\left(12.72\,{\text{D}}_{663}-2.59\,{\text{D}}_{645}\right)\,V\times N/M\times 1000$$

$$\mathrm{Chlorophyll}\,b=\,\left(22.88\,{\text{D}}_{645}-4.67\,{\text{D}}_{663}\right)\,\text{V}\times N/M\times 1000$$

Chlorophyll=⁢Chlorophyll⁢a+Chlorophyll⁢b

where *V* is the volume of photosynthetic pigment extract (ml), *W* is the sample (g), and *N* is the dilution factor.

### Leaf Nitrogen Content Measurement

*Toona sinensis* leaves were dried in a drying oven at 80°C for 48 h to constant weight and then ground with a ball mill. To determine the total nitrogen content, an appropriate amount of sample was taken with concentrated H_2_SO_4_-H_2_O for digestion, and a Kjeldahl nitrogen analyzer was used for automatic analysis (Horneck and Miller, 1997).

### Near Infrared Reflectance Spectroscopy Data Analysis

All modeling analyses were conducted in Rstudio (PBC, v4.0.4) (Team, 2020). The pipeline has two independent phases: (1) transformations and outlier detection and (2) model training and model selection (Yu et al., 2021). To correct the effects of light scattering or highlight the differences in absorption of light at different wavelengths, different spectral pretreatments, including standard normal variate (SNV), the first-order and the second-order differential with Savitzky–Golay smoothing along with their combinations were systematically applied to the averaged spectrum per sample (Alchanatis et al., 2005; Li et al., 2019).

The PLSR model (Hansen and Schjoerring, 2003) is a statistical linear method for fitting a curve by minimizing the sum of squared deviations, which combines the advantages of multiple linear regression, correlation analysis, and principal components. It is broadly applied in the near-infrared spectroscopy context (Li et al., 2021). Here, the samples were randomly split 100 times into calibration (80%) for model building and validation (20%) for testing. Four variable selection methods, the genetic algorithm PLS (GA), backward variable elimination PLS (Bve), significance multivariate correlation (sMC), and regularized elimination procedure in PLS (Rep), were used to extract important spectral feature variables from the preprocessed near-infrared spectral data (Shao et al., 2017; Liang et al., 2020). Each selection method was repeated 100 times. The selected model was then combined with the PLSR for prediction modeling of chlorophyll and nitrogen content.

To avoid the model overfitting, the number of latent variables of each PLS model have been set as less than 10 and the best latent variables number for each model has been selected use the one-sigma heuristic (Franklin, 2005) method. The evaluation of model performance was based on the calibrated correlation coefficient (*R*^2^_cal_), the root mean square error of the calibration set (RMSE_Cal_), the correlation coefficient of the validation set (*R*^2^_val_), the validation root mean square error set (RMSE_val_), residuals (R), and residual predictive deviation (RPD) (Hassan et al., 2015). Generally, a preferred model should have high values of *R*^2^_Cal_, *R*^2^_val_, and RPD, and lower RMSE_Cal_, RMSE_val_ values. The closer the *R*^2^ is to 1 with a RMSE and residuals close to 0, the better the prediction performance and stability of the model (Nicolai et al., 2007).

### Model Inversion

To obtain non-destructive dynamic monitoring of chlorophyll and N content in *T. sinensis* seedling leaves under various water conditions, one-way ANOVA was applied to examine the variations in chlorophyll and nitrogen of *T. sinensis* leaves under different drought stress treatments (Khaleghi et al., 2019). The variations between treatments were identified by using *post hoc* tests of Tukey’s honest significance difference (HSD).

## Results

### Statistics for Sampling Information and Data Preprocessing for Near-Infrared Spectroscopy

To establish a spectral prediction model with high prediction accuracy, three different varieties of *T. sinensis* seedlings under separate drought stress treatments were selected as sample sets. The chlorophyll and nitrogen content information of the collected *T. sinensis* seedlings is shown in Table 2. The large range of data ensures the robustness of the models derived from the data.

**TABLE 2:** Statistics of chlorophyll and nitrogen content of *T. sinensis* seedling leaves with different water gradients.

Treatment	Content	Max (mg/g)	Min (mg/g)	Mean (mg/g)	*SD*
100% FC	Chlorophyll	4.23	2.93	3.57	0.48
	Nitrogen	13.90	6.00	10.71	1.59
75% FC	Chlorophyll	4.05	3.16	3.19	0.52
	Nitrogen	14.5	6.50	10.73	1.59
50% FC	Chlorophyll	4.54	2.42	3.32	0.39
	Nitrogen	13.7	6.8	11.34	1.10
30% FC	Chlorophyll	4.55	2.53	3.39	0.51
	Nitrogen	14.80	8.10	11.54	1.11
10% FC	Chlorophyll	4.23	2.47	3.23	0.55
	Nitrogen	14.50	7.80	12.02	1.63

**FIGURE 1:**
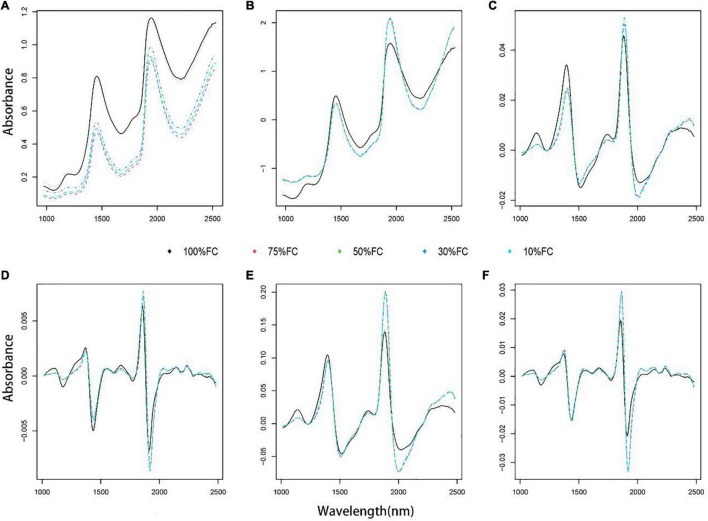
Original and various pretreatment spectra of *T. sinensis* seedling leaves collected under distinct water gradients. **(A)** Original spectra, **(B)** SNV: SNV spectra, **(C)** FSG: the first derivative combined with S-G smoothing spectra, **(D)** SSG: the second derivative combined with S-G smoothing spectra, **(E)** SNV_FSG: SNV combined with the first derivative and S-G smoothing spectra, **(F)** SNV_SSG: SNV combined with the second derivative and S-G smoothing spectra.

The original spectra of *T. sinensis* seedlings collected before different water treatments are shown in Figure 1A, which clearly illustrates that the original spectra of all samples show similar changes. The wavelengths have significant peaks between 1,400 ∼ 1,500 and 1,900 ∼ 2,000 nm (Figure 1A). Before modeling, five preprocessing methods were utilized to preprocess the spectrum, as shown in Figures 1B,F.

### Establishment and Optimization of a Near Infrared Spectroscopy Estimation Model of Chlorophyll and Nitrogen in *T. sinensis* Seedling Leaves

In the PLSR prediction models, the first derivative combined with SG smoothed spectrum (FSG) preprocessing predicted the best result for the chlorophyll and nitrogen content. Combining five spectral preprocessing methods with four variable selection methods for PLSR modeling significantly improved the accuracy of the model (mean chlorophyll and nitrogen *R*^2^_Cal_ were 0.71 and 0.62, respectively, with mean *R*^2^_val_ = 0.51 and 0.56, mean RMSE_Cal_ = 0.26 and 0.88, mean RMSE_val_ = 0.30 and 0.76; Figure 1). As a result, chlorophyll prediction showed *R*^2^_Cal_ = 0.73, with *R*^2^_val_ = 0.67, RMSE_Cal_ = 0.25, and RMSE_val_ = 0.26 (Supplementary Figure 3A).

Our modeling comparison showed that the prediction model established by using the SNV combined with the second derivative and SG smoothing spectra (SNV_SSG) preprocessing, as well as the Ga variable selection method, performed the best (Supplementary Figures 1, 3A). The mean *R*^2^_Cal_ = 0.72, *R*^2^_val_ = 0.51, RMSE_Cal_ = 0.25 and RMSE_val_ = 0.28, RPD = 2.26.

Nitrogen content prediction showed *R*^2^_Cal_ l and *R*^2^_val_ = 0.73 and 0.66, respectively, RMSE_Cal_ and RMSE_val_ = 0.85 and 0.71 (Supplementary Figure 3B). The prediction model established by using the first derivative combined with SG smoothing spectra (FSG) preprocessing and the sMC variable selection method again performed the best (Supplementary Figures 2, 3B) with *R*^2^_Cal_ = 0.63 (range between 0.60 and 0.66), *R*^2^_val_ = 0.52 (ranging from 0.46 to 0.57), RMSE_Cal_ = 0.87 and RMSE_val_ = 0.79, RPD = 1.12.

### Extraction of Characteristic Wavelength

**FIGURE 2:**
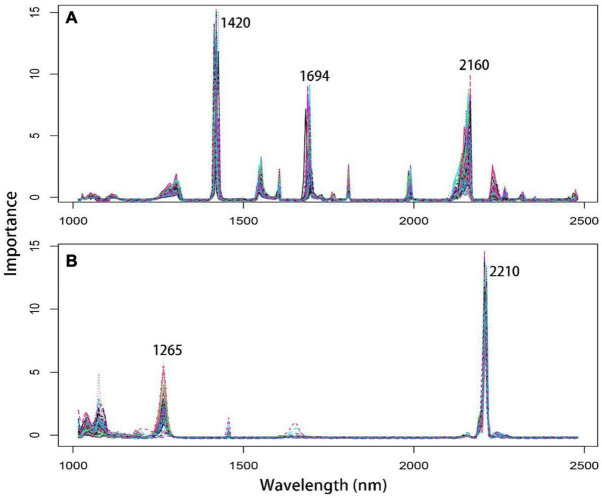
Characteristic wavelength extraction map of *T. sinensis* seedling leaves based on optimal spectral preprocessing and variable selection methods. **(A)** Chlorophyll content characteristic wavelength selection with the Ga variable selection method. **(B)** Nitrogen content characteristic wavelength selection with the sMC variable selection method.

The GA variable selection method found that the characteristic wavelengths of chlorophyll content were 1,420, 1,694 and 2,160 nm (Figure 2A). Among them, 1,420 nm was found to have the greatest influence on the prediction model, followed by 2,160 and 1,694 nm. Conversely, the N content prediction model only included two significant and important regions, 2,210 nm and 1,265 nm (Figure 2B).

### Comparisons of Optimal Model Results

**FIGURE 3:**
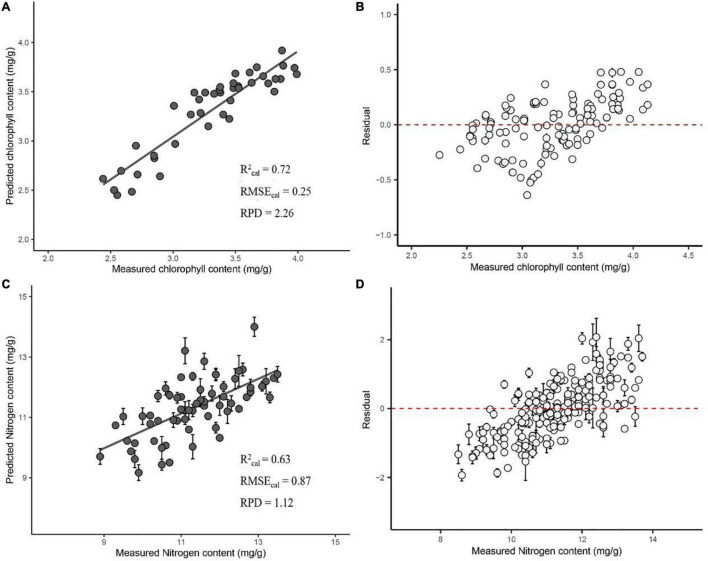
Optimal PLS model of the chlorophyll content based on SNV_SSG combined with the Ga variable selection method **(A)** and the nitrogen content based on FSG combined with the sMC variable selection method **(C)**. Residuals plotted of chlorophyll content **(B)** and nitrogen content **(D)**.

The residual value of the chlorophyll prediction model was between −0.5 and 0.5, indicating that the model had a good fitting outcome (Figures 3A,B). Conversely, the residual value of the N content prediction model was between −2 and 2, indicating a weaker performance (cv. chlorophyll content model; Figures 3C,D).

### Optimal Model to Predict the Chlorophyll and Nitrogen Contents of *T. sinensis* Leaves

**FIGURE 4:**
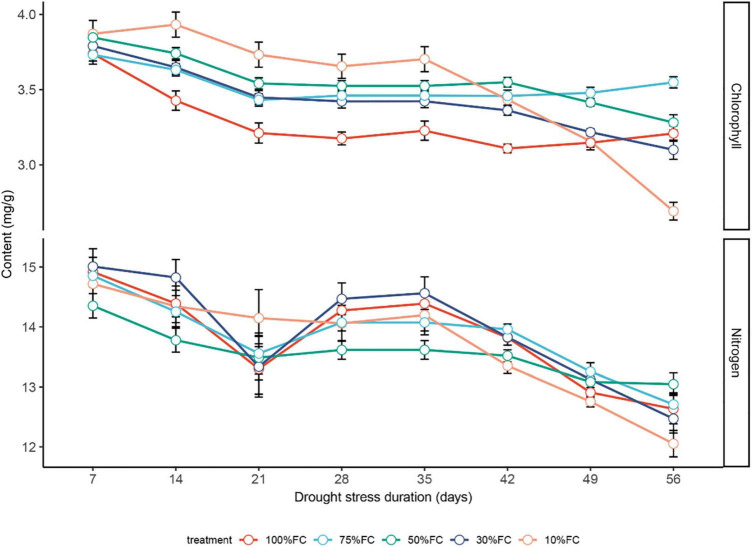
The effect of drought stress treatments on chlorophyll and nitrogen contents in leaves of *T. sinensis* leaves. The data represent the average of the spectrum inversion results under each treatment, and data are reported as the mean ± SE.

In general, under drought treatments, the chlorophyll content of *T. sinensis* leaves showed a decreasing trend over time. Among them, the chlorophyll content was the most stable under the 75% FC treatment, with the highest chlorophyll content on the 56th day (3.75 mg/g; Figure 4). The chlorophyll content of plant leaves under extreme water stress treatment (10% FC) treatment was significantly higher than other treatments in the first 35 days but dropped rapidly resulting in a significant lower value compared to the rest of other treatments (Figure 4). In the first week of the experiment, there was no divergence between different drought treatments.

**FIGURE 5:**
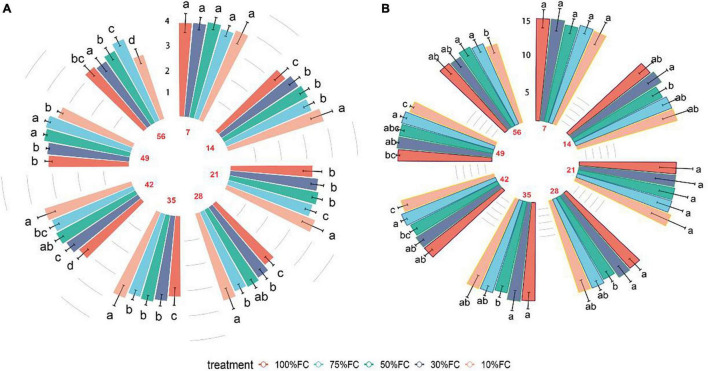
One-way ANOVA was used to examine the discrepancies between separate drought stress treatments over time. **(A)** Chlorophyll, **(B)** nitrogen. Vertical bars indicate ± SE. Comparison between treatments at the same time different letter indicates statistically significant differences according to Tukey’s HSD test. Values sharing a common letter are not significantly different at *p* < 0.01. Red numbers indicate the duration of drought stress.

Under different drought treatments, the leaf nitrogen content of *T. sinensis* seedlings changed over time, with a maximum increase in nitrogen content in the 10% FC treatment. The N content dropped at 35 and 56 days after the drought treatment (Figure 4). Overall, the chlorophyll and nitrogen content of plant leaves in the early period of drought were less affected by water. After 35 days, the extreme drought treatment (10% FC; Figures 5A,B) was significantly lower than other treatments, and the leaves began to yellow.

Under the three treatments with sufficient water (100% FC), severe stress (30% FC), and extremely severe stress (10% FC), the chlorophyll and nitrogen content of *T. sinensis* leaves showed a significant positive correlation. Under mild drought stress (75% FC), both chlorophyll and nitrogen were not significantly correlated.

## Discussion

Chlorophyll and nitrogen (N) content in *T. sinensis* were detected using five spectral preprocessing and four variable selection methods with a PLSR predictive modeling approach. The results showed that (1) chlorophyll content prediction was best determined by using SNV combined with the second derivative and SG smoothing spectra (SNV_SSG) preprocessing method as well as GA variable selection method; (2) N content prediction was determined by the first derivative combined with SG smoothing spectra (FSG) preprocessing method and sMC variable selection method. Overall, under various drought stress treatments, the *T. sinensis* leaf chlorophyll content showed a decreasing trend, with the most stable chlorophyll content under the 75% FC treatment. Conversely, the nitrogen content of the plant leaves showed a variable trend, and the nitrogen content decreased the most under the 10% FC treatment.

Due to the influence of environmental factors such as light conditions, the collected spectrum can contain more noise, which affects the construction of the spectrum model (Bobelyn et al., 2010). Spectral preprocessing effectively eliminates the influence of instrument noise (Martens et al., 1991). It has been reported that the choice of preprocessing method depends on the nature of the spectrum and the component characteristics that need to be predicted (Balabin et al., 2007). In our case, the original spectra of all samples showed similar content trends, but there were clear variations between the spectra for 100% FC and the other four treatments. This change may be related to the difference in the cell structure and optical propagation characteristics of *T. sinensis* leaves under various water treatments. At present, there are many kinds of spectral preprocessing methods, which can be divided into baseline correction, scatter correction, smoothing, etc. according to the effect of preprocessing (Li et al., 2019, 2021). Baseline correction includes first-order and second-order derivative, etc.; scattering correction includes multiplicative scatter correction (MSC), SNV, etc.; smoothing includes S-G smoothing, etc. (Nicolai et al., 2007; Liang et al., 2020). Among them, the derivative processing is mainly to deduct the influence of instrument background or drift on the signal; MSC and SNV are used to eliminate the influence of scattering on the spectrum due to uneven particle distribution and different particle sizes; S-G smoothing can very effectively improve the spectral information, reduce the influence of random noise (Hassan et al., 2015). Therefore, combining different preprocessing methods was beneficial to improve the accuracy of the model. The results showed that different preprocessing methods reduced the spectral signal-to-noise ratio (SNR) to various degrees and improved the accuracy. Compared with the other four processing methods, the standard normal variable (SNV) focuses on baseline removal, and the spectral smoothing result is weak (Figure 1B). This confirms that equipment, range, environment, and other spectrometer factors affect the preprocessing spectral results. The combination of multiple preprocessing methods determined a high accuracy, which is conducive to constructing the best model performance. Variable selection instead reduces the number of irrelevant variables, which may contain noise and outliers, therefore significantly improving the sensor performance (Prananto et al., 2020).

Previous studies such as Cozzolino (2015) showed that plant pigments and phytonutrients in the form of organic matter are directly measured with near-infrared spectroscopy because these compounds contain chemical bonds that are identified in signal peaks in the NIRS, and the compound abundance is correlated with the intensity of those specific peaks. Furthermore, Lee et al. (2000) found that chlorophyll has a strong absorption value in the visible and NIRS produced by the conjugated C–C and C = C bonds of the porphyrin ring and magnesium (Mg) ions. Moreover, Kokaly (2001) also reported, with high accuracy, NIRS regions related to the chlorophyll contents [1768, 1818, 1850, 2076, 2304, and 2350 nm; cv. (Leon-Saval et al., 2004)]. Min and Lee (2005) studied citrus leaves and found that significant wavelengths for chlorophyll detection were 448, 669, 719, 1,377, 1,773, and 2,231 nm. In our case, the most significant chlorophyll bands were at 1,420, 1,694, and 2,160 nm, indicating strong light absorbance by chlorophyll content at these bands.

N is an important component of chlorophyll and protein (Min et al., 2006). The C–H and N–H bonds contained in the cells are detected in the NIRS (Shao and He, 2013). The protein has a significant influence on the NIRS in the 2,172–2,054 nm wavelength range (Min and Lee, 2005). Similarly, our study found that the N content was most significant at 2,210 nm, confirming the accuracy of our variable selection method.

Water absorbs light in the near-infrared range. Experiments conducted by Curcio and Petty (1951) showed that water has prominent absorption bands at wavelengths of 760, 970, 1,190, 1,450, and 1,940 nm. When the water content in plants is distinguishable, the reflectance of the visible light and near-infrared spectral regions are also different. Experimental studies have found that when the water content of plants is reduced to 50%, the spectral reflection speed increases significantly (Carter and Knapp, 2001). Therefore, the moisture in the leaves may affect the spectral absorption to a certain extent, affecting the prediction model fitting. In our study, *T. sinensis* seedling leaves selected during spectrum collection were fresh samples containing water. In this context, the prediction models for chlorophyll and nitrogen content had *R*^2^ = 0.72 and 0.62, respectively, indicating good predictive accuracy but still to be improved. Future research should further consider the influence of factors such as moisture to improve model performance.

Chlorophyll content is an important evaluation index of plant responses to drought stress (Hassan et al., 2015). Generally, drought stress not only affects chlorophyll content production but also reduces chlorophyll storage capacity (Kuroda et al., 1990). Majumdar et al. (1991) reported that chlorophyll was reduced under drought stress, similar to our results.

**FIGURE 6:**
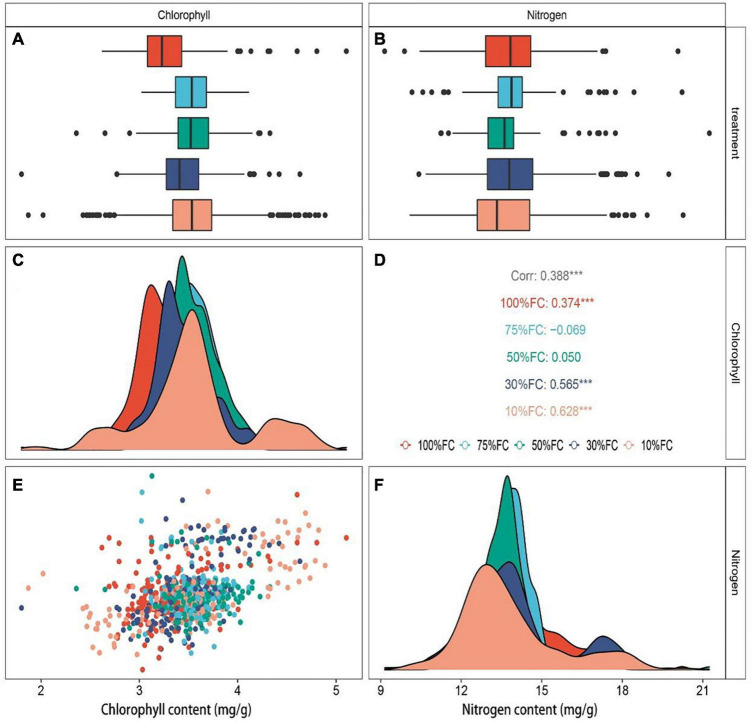
Chlorophyll **(A)** and nitrogen **(B)** content under separate drought treatments. Correlation analysis of chlorophyll and nitrogen content of *T. sinensis* leaves under separate drought treatments **(D)**. Chlorophyll **(C)** and nitrogen **(F)** content were normally distributed. Distribution map of chloroplast content of all samples **(E)**. Statistical significance was determined by two-tailed *t*-test with equal variance comparing the unique drought treatments of chlorophyll and nitrogen content; ***p < 0.001. Different colors indicate separate treatments, and the corresponding colors are the same as the above figure.

Several studies have reported a negative impact on plant nutrient absorption by the intensification of drought stress (He and Dijkstra, 2014). For example, the ecological and physiological responses of *Abies fabri* seedlings to drought stress and nitrogen supply have been reported (Guo et al., 2010). Similar to our study, the nitrogen content of *T. sinensis* leaves showed a variable trend. Due to the limited water supply, the reduction of plant leaf stomatal conductance and carbon (C) assimilation hinds the migration of nitrogen and other nutrients in the leaf (Bänziger et al., 1999; He and Dijkstra, 2014). This is in line with our findings at 21 days (Figure 6). It has also been supported that drought stress increases the content of malondialdehyde (MDA), proline, soluble sugar and other substances, and the nitrogen supply in leaves alleviates the effects of drought stress on plants (He and Dijkstra, 2014). Because the chlorophyll and nitrogen contents in leaves are affected by many factors, real-time and dynamic detection of their contents can help to take early proactive measures.

Nitrogen supply has a strong influence on leaf growth (He and Dijkstra, 2014). Plant leaf area growth promotes photosynthesis at the same time (Baresel et al., 2017). Chlorophyll is the main product of photosynthesis. Thus, chlorophyll content is also approximately proportional to leaf nitrogen content (Bojović and Marković, 2009). Similarly, our study found that the optimal prediction model based on NIRS technology can predict chlorophyll and nitrogen content in leaves in a non-destructive dynamic monitoring way.

## Conclusion

Our study has shown that NIRS have great potential in field applications for the analysis of chlorophyll, nitrogen and other elements in plant leaf tissues and can achieve a non-destructive dynamic monitoring effect.

## Data Availability Statement

The original contributions presented in the study are included in the article/Supplementary Material, further inquiries can be directed to the corresponding author.

## Author Contributions

YL and WL conceived the ideas and designed the methodology. WL collected and analyzed the data and wrote the manuscript. YL guided the data analysis and reviewed the manuscripts. FT and WY revised the content and grammar of the manuscript. JL, JJ, and ZT supervised all stages of the experiments. All authors read and approved the final manuscript.

## Conflict of Interest

FT was employed by company AgResearch Ltd. The remaining authors declare that the research was conducted in the absence of any commercial or financial relationships that could be construed as a potential conflict of interest.

## Publisher’s Note

All claims expressed in this article are solely those of the authors and do not necessarily represent those of their affiliated organizations, or those of the publisher, the editors and the reviewers. Any product that may be evaluated in this article, or claim that may be made by its manufacturer, is not guaranteed or endorsed by the publisher.

## Supplementary Material

Supplementary Figure 1 Linear fitting graph of PLS modeling results of *T. sinensis* seedling chlorophyll content based on five spectral preprocessing and four variable selection methods. Red represents the optimal model for predicting.

Supplementary Figure 2 Linear fitting graph of PLS modeling results of *T. sinensis* seedling Nitrogen content based on five spectral preprocessing and four variable selection methods. Blue represents the optimal model for predicting.

Supplementary Figure 3 PLS modeling results of chlorophyll **(A)** and nitrogen **(B)** content of *T. sinensis* seedlings based on five spectral preprocessing and four variable selection methods. R2cal: corrected correlation coefficient, R2val: verified correlation coefficient, RMSEcal: corrected root mean square error, RMSEval: verified root mean square error. Red represents the optimal model for predicting chlorophyll content, and blue represents the optimal model for predicting nitrogen content.
