# Supplementary figures and images for: Non-destructive Measurements of Toona sinensis Chlorophyll and Nitrogen Content Under Drought Stress Using Near Infrared Spectroscopy
Source: Front Plant Sci. 2022 Jan 21;12:809828. doi: 10.3389/fpls.2021.809828 (PMC8814108; doi:10.3389/fpls.2021.809828)

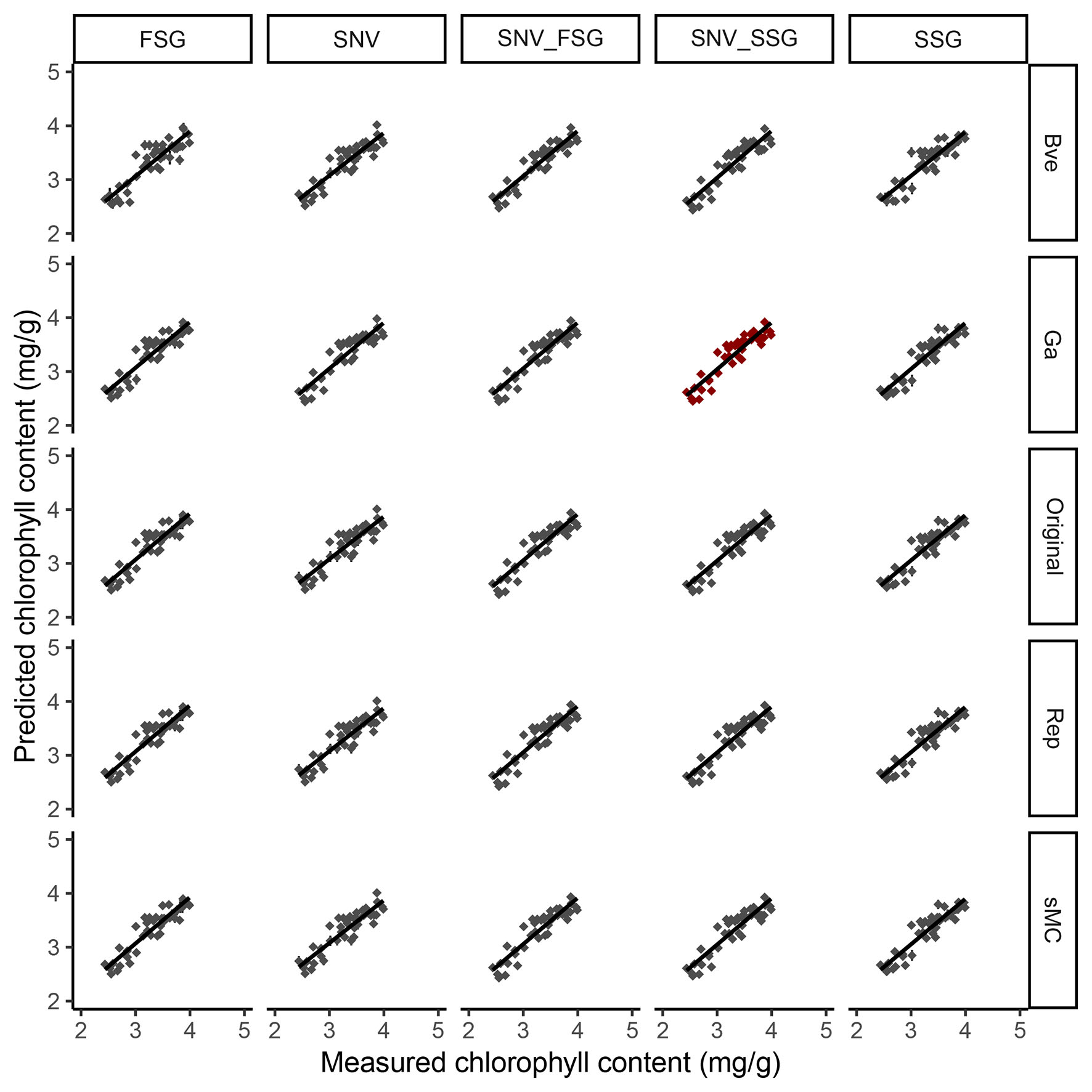

Supplement: Supplementary Figure 1 — Linear fitting graph of PLS modeling results of T. sinensis seedling chlorophyll content based on five spectral preprocessing and four variable selection methods. Red represents the optimal model for predicting. [file Image_1.jpg]

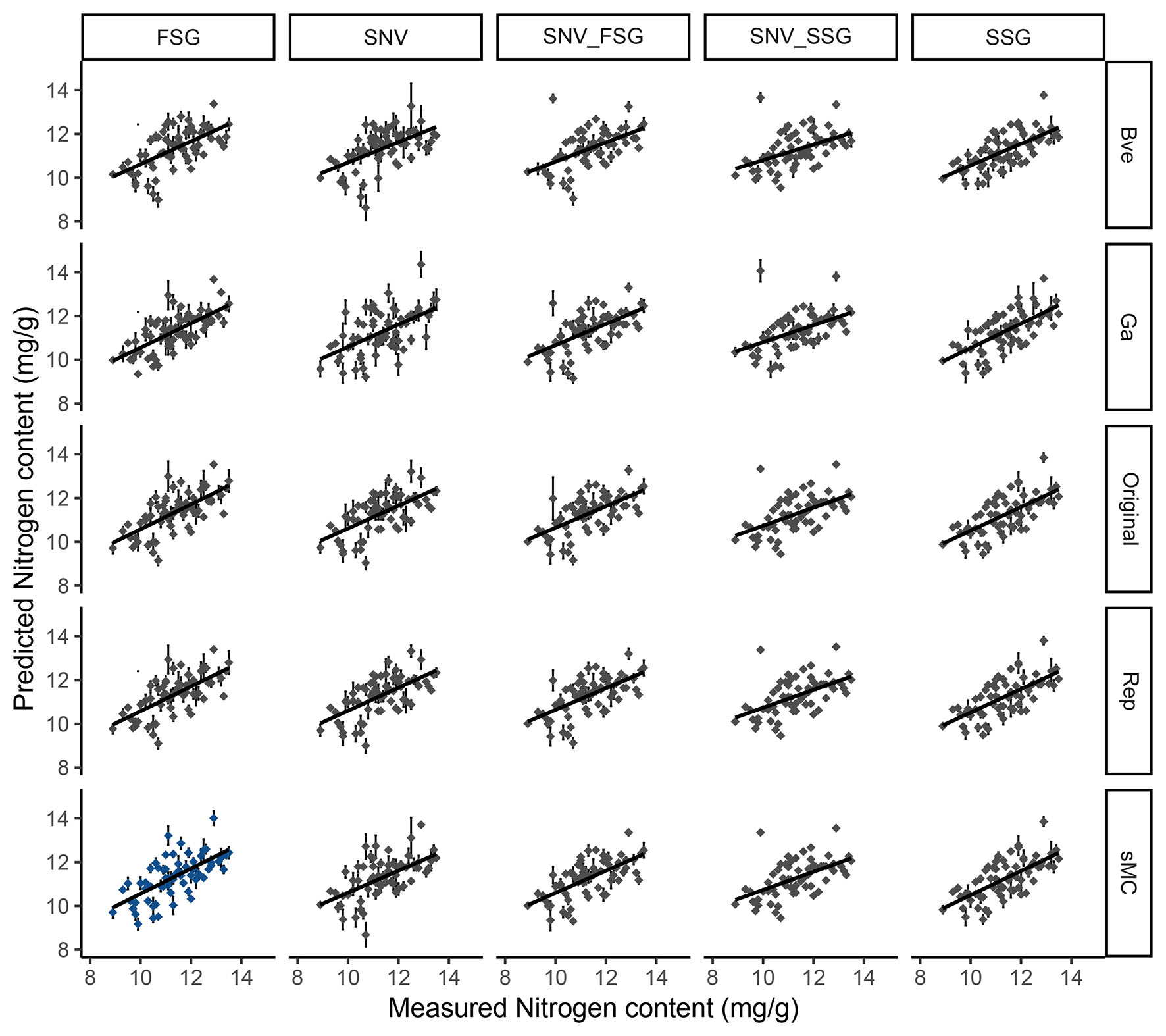

Supplement: Supplementary Figure 2 — Linear fitting graph of PLS modeling results of T. sinensis seedling Nitrogen content based on five spectral preprocessing and four variable selection methods. Blue represents the optimal model for predicting. [file Image_2.jpg]

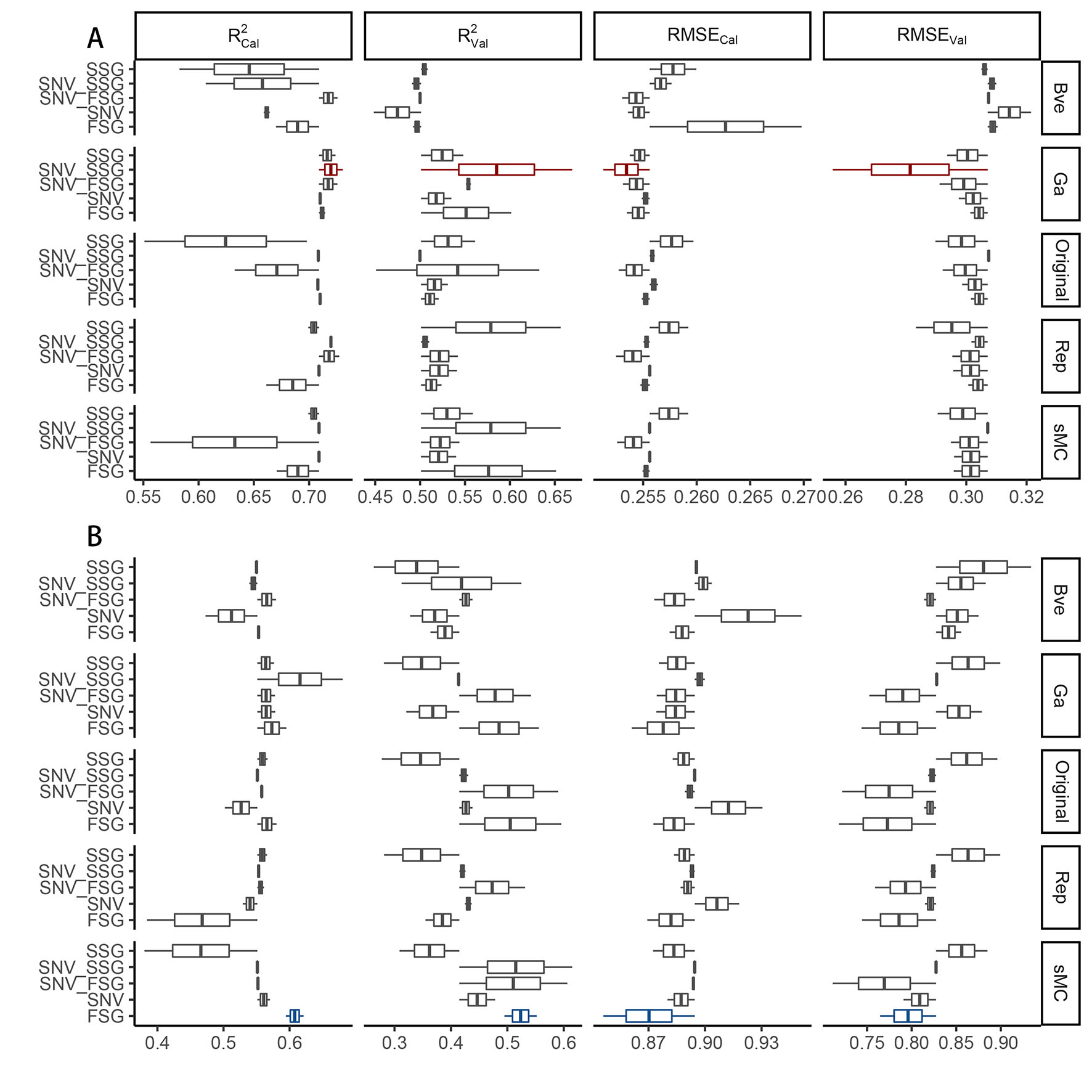

Supplement: Supplementary Figure 3 — PLS modeling results of chlorophyll (A) and nitrogen (B) content of T. sinensis seedlings based on five spectral preprocessing and four variable selection methods. R2cal: corrected correlation coefficient, R2val: verified correlation coefficient, RMSEcal: corrected root mean square error, RMSEval: verified root mean square error. Red represents the optimal model for predicting chlorophyll content, and blue represents the optimal model for predicting nitrogen content. [file Image_3.jpg]
